# The feasibility of malaria elimination in South Africa

**DOI:** 10.1186/1475-2875-11-423

**Published:** 2012-12-19

**Authors:** Rajendra Maharaj, Natashia Morris, Ishen Seocharan, Philip Kruger, Devanand Moonasar, Aaron Mabuza, Eric Raswiswi, Jaishree Raman

**Affiliations:** 1 Malaria Research Unit, Medical Research Council, 491 Ridge Road, Durban, KwaZulu-Natal 4001, South Africa; 2 Department of Health and Social Welfare, Limpopo Provincial Government, PO Box 33, Tzaneen, 0850, South Africa; 3 National Department of Health, 1916 Hallmark Building, Private Bag X828, Pretoria, 001, South Africa; 4 Department of Health and Social Services, Mpumalanga Provincial Government, Private Bag X11278, Nelspruit, Mpumalanga, 1200, South Africa; 5 Department of Health-KwaZulu-Natal, KwaZulu-Natal Provincial Government, Private Bag X002, Jozini, KwaZulu-Natal, 3969, South Africa

**Keywords:** Malaria elimination, Feasibility, South Africa, Vector control, Case management, Surveillance

## Abstract

**Background:**

Following the last major malaria epidemic in 2000, malaria incidence in South Africa has declined markedly. The decrease has been so emphatic that South Africa now meets the World Health Organization (WHO) threshold for malaria elimination. Given the Millennium Development Goal of reversing the spread of malaria by 2015, South Africa is being urged to adopt an elimination agenda. This study aimed to determine the appropriateness of implementing a malaria elimination programme in present day South Africa.

**Methods:**

An assessment of the progress made by South Africa in terms of implementing an integrated malaria control programme across the three malaria-endemic provinces was undertaken. Vector control and case management data were analysed from the period of 2000 until 2011.

**Results:**

Both malaria-related morbidity and mortality have decreased significantly across all three malaria-endemic provinces since 2000. The greatest decline was seen in KwaZulu-Natal where cases decreased from 42,276 in 2000 to 380 in 2010 and deaths dropped from 122 in 2000 to six in 2010. Although there has been a 49.2 % (8,553 *vs* 4,214) decrease in the malaria cases reported in Limpopo Province, currently it is the largest contributor to the malaria incidence in South Africa. Despite all three provinces reporting average insecticide spray coverage of over 80%, malaria incidence in both Mpumalanga and Limpopo remains above the elimination threshold. Locally transmitted case numbers have declined in all three malaria provinces but imported case numbers have been increasing. Knowledge gaps in vector distribution, insecticide resistance status and drug usage were also identified.

**Conclusions:**

Malaria elimination in South Africa is a realistic possibility if certain criteria are met. Firstly, there must be continued support for the existing malaria control programmes to ensure the gains made are sustained. Secondly, cross border malaria control initiatives with neighbouring countries must be strongly encouraged and supported to reduce malaria in the region and the importation of malaria into South Africa. Thirdly, operational research, particularly on vector distribution and insecticide resistance status must be conducted as a matter of urgency, and finally, the surveillance systems must be refined to ensure the information required to inform an elimination agenda are routinely collected.

## Background

Malaria elimination is rapidly becoming a tempting alternative to malaria control in many malaria-endemic African countries [1]. This paradigm shift is largely a consequence of effective malaria control initiatives, which have decreased markedly the malaria burden across the African continent [2]. Countries along the southernmost fringe of malaria transmission, such as Angola, Botswana, Swaziland and South Africa, already meet the WHO pre-elimination criteria and have therefore been earmarked for malaria elimination by 2020. Swaziland embarked on an elimination campaign in 2011 [3], while South Africa is preparing to implement its elimination strategy during the 2012/2013 malaria season.

Currently in South Africa, malaria is restricted to low-altitude border regions (below 1,000 m above sea level) of three provinces, namely Limpopo, Mpumalanga and KwaZulu-Natal, with limited transmission along the Molopo and Orange Rivers in the North West and Northern Cape Provinces, respectively. Approximately 10% (4.9 million) of South Africa’s total population resides in a malaria risk area [4], with the predominant malaria parasite, *Plasmodium falciparum,* primarily transmitted by the *Anopheles arabiensis* mosquito vector. Malaria transmission is meso-endemic, occurring between September and May but peaking in March.

Following the last major epidemic in 2000, where more than 60,000 malaria cases were reported, a two-pronged intervention, focussing on both the vector and parasite, was implemented across all three malaria-endemic provinces. Indoor residual spraying (IRS) formed the principal vector control measure while timely diagnosis and effective treatment with artemisinin-based combination therapy (ACT) were used to control the parasite. These effective, well-structured, sustainable control strategies have resulted in marked reductions in the malaria burden, to the extent that the current malaria incidence in South Africa is less than one case per 1,000 population at risk.

This seemly low malaria incidence has prompted international and governmental organizations to call for the urgent adoption and implementation of elimination agenda by South Africa. Unfortunately these calls have generally been based solely on annual incidence data rather than a rigorous interrogation of the available scientific data. As the consequences of failure are likely to be costly in monetary terms and more importantly with respect to the loss of human life, the decision to move from control to elimination should not be taken lightly. It is therefore essential the appropriateness, timing as well as technical, operational and financial feasibility of implementation are thoroughly assess prior to embarking upon an elimination programme [5].

The World Health Organisation (WHO) recommends major programmatic reorientation occurs when transitioning from malaria control to elimination. High coverage interventions must become more geographically targeted while laboratory and clinical services together with case reporting and surveillance are substantially up-scaled [6]. This study aimed to determine the readiness, appropriateness as well as technical and operational feasibility of implementing an elimination programme in present day South Africa. The factors assessed included numbers of malaria cases and deaths, IRS coverage rates, levels of resistance to insecticides used for IRS, vector distribution, prevalence of anti-malarial resistance markers and the efficiency of the malaria information system (MIS). It is hoped data from this study will be used to inform the current South African malaria elimination agenda.

## Methods

### Morbidity and mortality data

Malaria cases confirmed at health facilities across the three South African malaria-endemic provinces are entered into a clinic or hospital case register and reported telephonically to the district health office. Individual case records are also routinely entered onto malaria notification forms, which are submitted on a weekly basis to provincial malaria control programmes (MCP). At the MCP offices, individual case data including patient details, symptoms, diagnosis, microscopy and/or rapid diagnostic test (RDT) results, treatment administered, referrals information, the locality the patient resides in and the reporting health facility’s name are entered onto a computerized malaria information system (MIS) developed using Microsoft products [7]. Case reports generated by the MIS are then issued to field surveillance agents for follow-up and investigation. Upon conclusion of field surveillance, case follow-up forms are returned to the provincial MCP where any new information (including treatment outcome and potential source of infection) obtained is entered into the MIS using the unique case number to ensure the new data can be linked to the original patient record. The MIS allows for data entry, querying and reporting at the individual patient level or as spatial and temporal aggregates.

### Incidence calculation and determination of elimination status of provinces

A patient’s place of residence, rather than source location of infection, were used to calculate malaria case aggregates and malaria incidence as these data are routinely collected by health facilities. Source of infection is generally determined during a follow-up visit to the patient’s home by a case investigation officer. During these visits patient are questioned on recent travel activities, previous malaria history as well possible contact with malaria vectors and/or malaria patients. Unfortunately as a relatively high proportion of patients were not located during follow-up investigations, this data are not available for all reported malaria cases. Over the study period, source data from 26% and 27% of the case data from KwaZulu-Natal and Limpopo respectively were missing. In contrast, the source of infection for all reported cases in Mpumalanga was captured by the MIS. A case was classified as imported if the patient had travelled to a malaria endemic area in the past month and/or if there was no evidence of local transmission (lack of vectors and other malaria cases within a 500 meter radius of the index malaria case).

The administrative construct of municipality or sub-district was selected as the appropriate level of classification in order to provide clear insights into the degree of variation that is evident in the national situation. Patient residence data from provincial MISs were used to generate malaria case aggregates per municipality for each malaria endemic province by malaria season, running from July of one year to June of the following year.

Population data stored within the MIS, sourced from the South African National Population Census of 2001 [8] and the National Community Survey of 2007 [9], were used to calculate malaria incidence for each municipality. For non-census years, population data were estimated using the annual incremental growth rate observed between the population census and survey years and then used to calculate municipal-level malaria incidence for each of the malaria seasons.

The WHO defines four key phases along the path a malaria programme will follow towards the goal of eventual elimination, and recommends systematic programme reorientation at each stage to increase success rates in transitioning to the next [6]. The resulting incidence values were used to classify individual municipalities into one of the four WHO defined phases of the malaria elimination continuum, namely malaria control (incidence >five malaria cases per 1,000 population at risk), pre-elimination (incidence between zero and five malaria cases per 1,000 population at risk), elimination (incidence <one malaria case per 1,000 population at risk) and prevention of reintroduction (zero malaria cases per 1,000 population at risk) per malaria season [6].

The calculation of incidence and associated malaria elimination status using patient residential data were further qualified by the proportion of local cases observed, where municipal areas with no local cases reported in the previous five years were considered to be in the prevention phase. Map output was generated using ArcGIS ^®^ software [10].

### Indoor residual spraying

Indoor residual spraying (IRS) operations are conducted in the three South African malarious provinces during the malaria season, using dichloro-diphenyl-trichloro-ethane (DDT), carbamates or pyrethroid insecticides. Spray officers spray each room of a homestead in areas under active malaria control and surveillance. On completion of a single spray session, records of the activities conducted are entered onto a spray card. These are then submitted to the IRS team leader who verifies data quality and completeness prior to forwarding the card to the provincial MCP. At the MCP offices the spray data are captured into a customized spraying information system (SIS). This application allows for data entry, querying and reporting. Managers are able to monitor spray progress, spray coverage, performance of individual spray officers, insecticide usage, and application rates via predefined reports in the SIS [11]. Spray data are only available from 2005 onwards as the SIS was implemented countrywide in that year. Prior to SIS implementation IRS data were collected in disparate formats.

### Anti-malarial resistance marker prevalence

Parasite DNA was extracted from the positive malaria rapid test kits (ICT™, Global Diagnostics, Cape Town, South Africa; First Response™, Premier Medical Corporation Ltd, Kachigan, Daman, India) using the Chelex method [12]. Once a sample was confirmed as *P. falciparum* positive by qPCR [13], mutational analysis was conducted to detect molecular markers associated with chloroquine (*crt*K76T and *mdr1*N86Y) and sulphadoxine-pyrimethamine (SP, *dhfr*N51I, *dhfr*C59R, *dhfr*S108N, *dhfr*I164L, *dhps*S436A, *dhps*A437G, *dhps*K540E and *dhps*A581G) resistance. Primers, PCR amplification conditions and restriction endonucleases used to detect polymorphisms in the genes of interest have been described previously [14-16]. Digestion products separated on a 2% agarose gel using electrophoresis were visualized and photographed using a MiniBIS™ documentation system (BioSystematica, UK). Codons were classified as either pure sensitive, pure mutant or mixed (both mutant and sensitive genotypes present in an individual sample). Genotyping was run in duplicate, with a third assay being performed on any discordant results. When calculating overall prevalence of infections with mutant genotypes, codons with mixed genotypes were grouped with pure mutant codons. Copy number of the *mdr1* gene associated with lumefrantrine was assessed using the qPCR method, primers, probes and qPCR cycling conditions previously described by Price *et al.*[17]. Every qPCR run contained reference DNA samples from D10 and Fac8 clones, with *mdr1* copy numbers of 1 and 3 respectively.

### Availability of rapid diagnostic tests and artemisinin-based combination therapy

According to South Africa’s national malaria treatment guidelines, all suspected malaria cases must be confirmed either by RDT or microscopy prior to treatment with the artemisinin combination, artemether-lumefantrine, marketed as Coartem^®^. As numbers of RDTs used and anti-malarials dispensed were not captured by the MIS or pharmaceutical depots, a literature survey was carried out to obtain this information.

### Insecticide resistance data

Insecticide resistance data have not been routinely collected by the provincial MCP, so a literature search was conducted to determine the most current insecticide resistance profile for South Africa.

## Results

### Malaria morbidity and mortality

#### Historical trends

**Figure 1:**
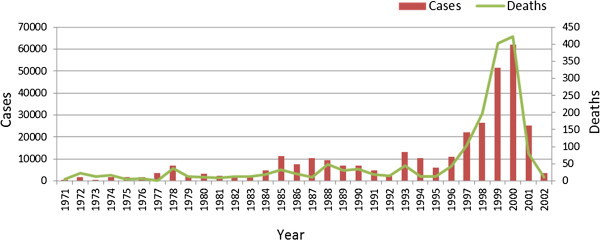
Number of malaria cases and deaths reported annually in South Africa (1971–2002).

Malaria case numbers and deaths have been well documented at a national level since 1971 (Figure 1). Between 1971 and 1996, annual malaria case numbers very rarely exceeded 10,000 cases. However in 1997 case numbers began increasing sharply, peaking at over 60,000 cases in 2000. Following changes in insecticide and anti-malarial policy, case number dropped to well below 10,000. Epidemic years reported by the Department of Health occurred in 1972, 1978, 1985, 1993 and most recently in 2000. The mortality data mirrors the morbidity data trend (Figure 1).

#### Current trends

**Figure 2:**
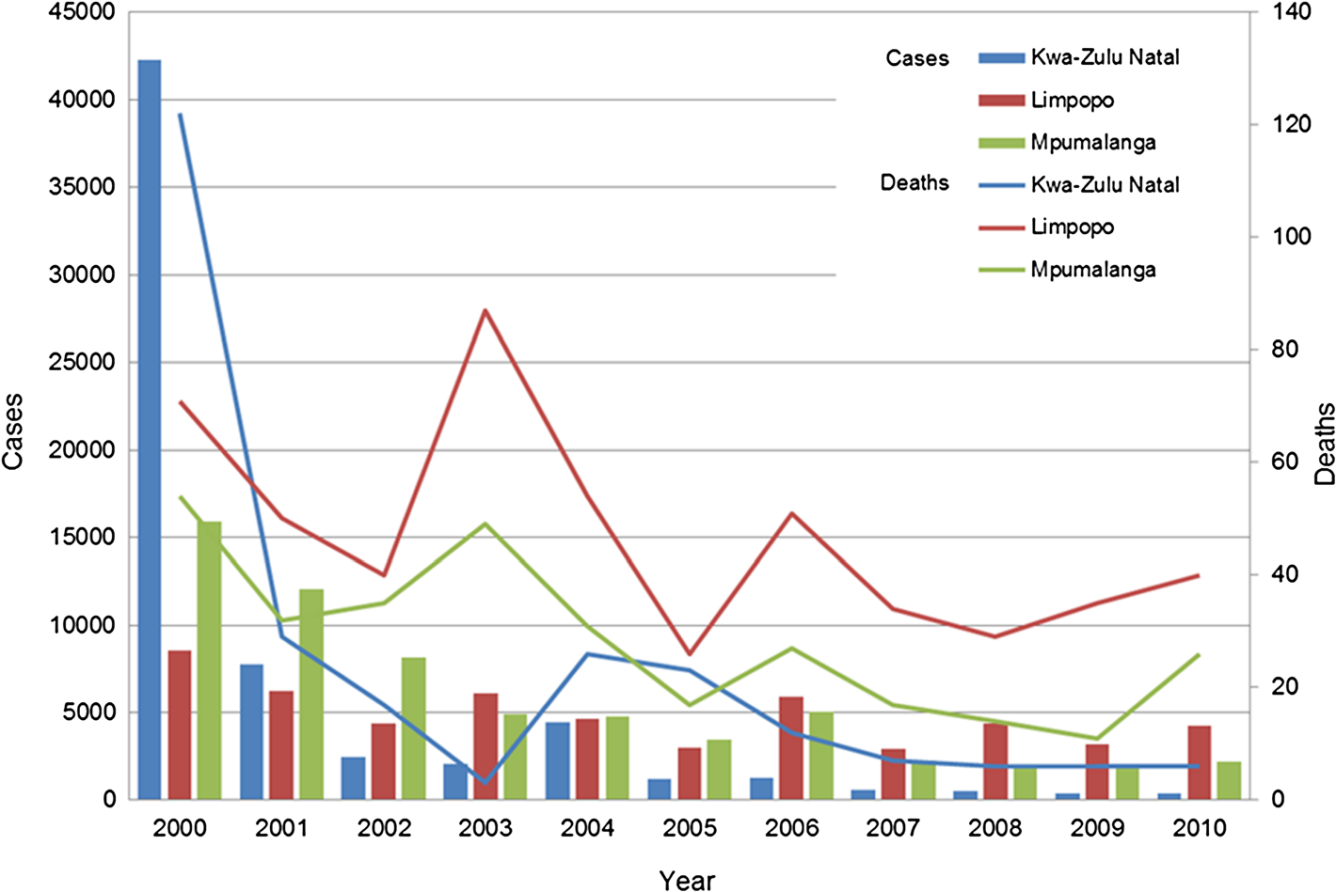
Numbers of malaria cases and malaria deaths by year and malaria-endemic province.

Over the 10-year study period, the total number of reported malaria cases decreased markedly from 66,700 in 2000 to 6,788 in 2010 (Figure 2). The largest decrease was noted in KwaZulu-Natal, with this province reporting the lowest number of cases in 2010 (Figure 2). Although case numbers in Limpopo declined from 8,553 in 2000 to 4,214 in 2010, this province has become the largest contributor to malaria incidence of the three endemic provinces (Figure 2).

Similar to malaria case numbers, malaria-related mortality decreased during the study period, from 247 deaths in 2000 to 72 deaths in 2010 (Figure 2). Again, the province associated with the greatest decline in malaria-associated deaths was KwaZulu-Natal, while the highest number of deaths in 2010 was reported in Limpopo (Figure 2).

#### Imported malaria

The proportion of locally acquired infections compared to those originating from neighbouring countries differs among the three malaria-endemic provinces (Table 1). In both KwaZulu-Natal and Mpumalanga imported case numbers far exceed locally acquired cases numbers. Limpopo was the only province where no pronounced decrease in locally acquired cases was noted. There are also high percentages of unclassified cases in both KwaZulu-Natal and Limpopo that may be biasing the results.

**Table 1:** Imported and local malaria cases reported in KwaZulu-Natal, Limpopo and Mpumalanga Provinces, South Africa (2005–2011)

**Province**	**Malaria Season**	**Local (%)**	**Imported (%)**	**Unclassified (%)**
KwaZulu-Natal	2005/2006	30	41	29
	2006/2007	20	46	35
	2007/2008	14	52	34
	2008/2009	11	47	41
	2009/2010	11	42	48
	2010/2011	19	50	31
Limpopo	2005/2006	60	13	27
	2006/2007	71	14	15
	2007/2008	73	10	17
	2008/2009	52	14	34
	2009/2010	62	15	23
	2010/2011	57	18	24
Mpumalanga	2005/2006	31	60	9
	2006/2007	32	68	0
	2007/2008	30	70	0
	2008/2009	25	75	0
	2009/2010	28	72	0
	2010/2011	20	80	0

#### Malaria elimination status of endemic provinces

**Figure 3:**
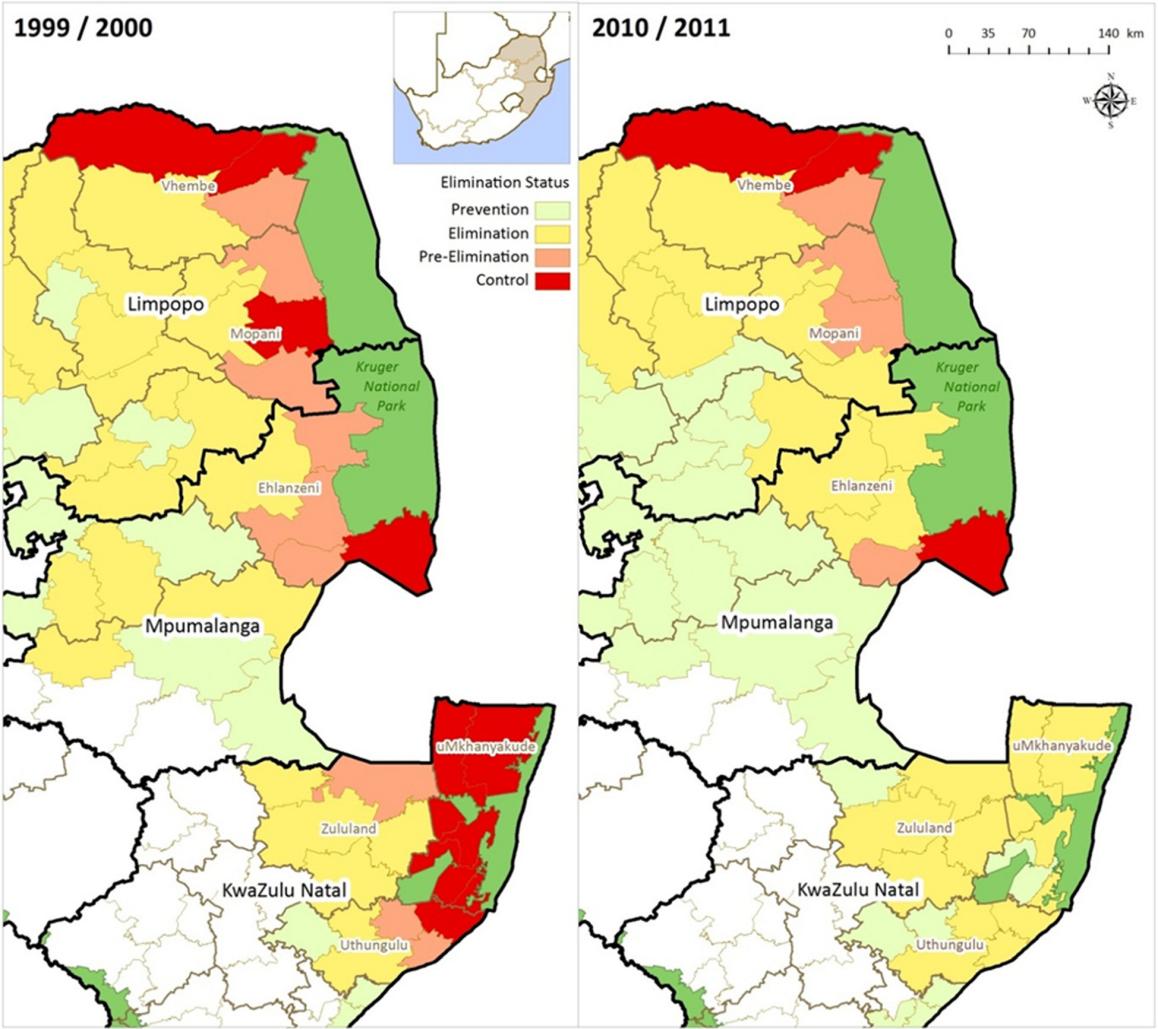
Phase of malaria elimination continuum per district in KwaZulu-Natal, Limpopo and Mpumalanga, South Africa (1999/2000 and 2010/2011 malaria seasons).

All three malaria-endemic provinces are at different phases within the malaria elimination continuum (Figure 3). All districts within KwaZulu-Natal have an incidence of <one malaria case per 1,000 population at risk and therefore this province as a whole meets the WHO criteria to move from a control phase to an elimination phase. Although areas of active transmission in Mpumalanga have decreased greatly, two municipal areas within the Ehlanzeni District (Figure 3) have malaria incidences that exceed the elimination phase threshold. One area remains in the control phase while the second falls into the pre-elimination category (Figure 3).

Malaria incidence in Limpopo has altered minimally over the study period (Figure 3). The high-risk area in Vhembe District has not changed substantially since the 1999/2000 season and remains in the control phase. According to the 1999/2000 malaria incidence map, two of the five municipalities in Mopani District were in the pre-elimination phase, one was in the control phase while the remaining two areas were already in the elimination phase. In the 2010/2011 map, the two areas in the elimination phase remain unchanged, while the area in the control phase has been elevated to pre-elimination status with one of the 1999/2000 pre-elimination areas gaining an elimination rating.

#### Indoor residual spraying coverage per province

**Figure 4:**
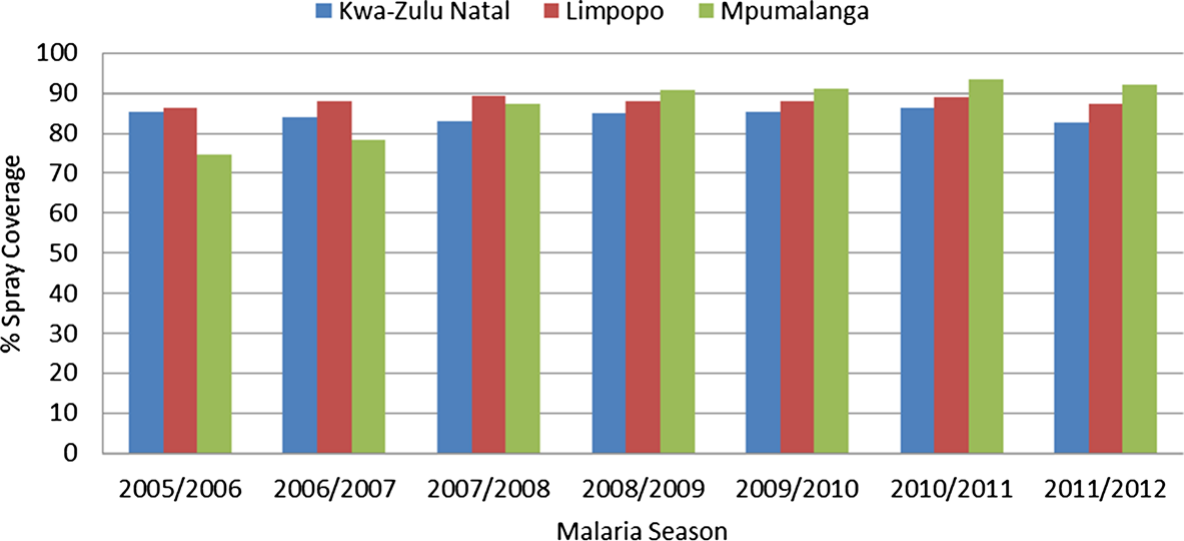
Indoor residual spraying coverage in KwaZulu-Natal, Limpopo and Mpumalanga, South Africa (2005/2006 and 2010/2011 malaria seasons).

Over the study period, residual insecticide spray coverage rates in all three malaria-endemic provinces exceeded 70% (Figure 4). During the 2005/2006 malaria season, spray coverage rates in both KwaZulu-Natal and Limpopo surpassed the 80% minimum cut-off limit, while coverage in Mpumalanga fell below this limit (Figure 4). Coverage in Mpumalanga increased over the following six spray seasons, reaching 92% by 2010/2011. Although KwaZulu-Natal maintained a spray coverage rate greater than 80% during the study period, in the 2010/2011 season it was the poorest performing, reporting 82% coverage compared with 87% and 92% in Limpopo and Mpumalanga respectively (Figure 4).

#### Insecticide resistance

A literature survey revealed a severe dearth in insecticide resistance data for South Africa (Table 2). Only three published reports were found, all from KwaZulu-Natal, with two studies on *Anopheles funestus* and one on *An. arabiensis* (Table 2). The main vector in KwaZulu-Natal, *An. arabiensis*, was found to be resistant to DDT but sensitive to pyrethroids [18]. In contrast the *An. funestus* vector was found to be resistant to pyrethroids [19] but sensitive to DDT and carbamates [20].

**Table 2:** Published reports of insecticide resistance in KwaZulu-Natal, Limpopo and Mpumalanga, South Africa

**Insecticide**		**Province**	
	**KwaZulu-Natal**	**Limpopo**	**Mpumalanga**
DDT	*An. arabiensis*[18]	No data	No data
Pyrethroid	*An. funestus*[19]	No data	No data
Carbamate	*An. funestus*[20]	No data	No data
Organophosphate	No data	No data	No data

### Rapid diagnostic tests and artemisinin-based combination therapy

The literature review revealed that currently all malaria cases are being definitely diagnosed by RDT or blood smear. The uptake of ACT was 172% in 2009 [2] improving to 100% by 2011 [21].

#### Anti-malarial resistance marker prevalence

**Figure 5:**
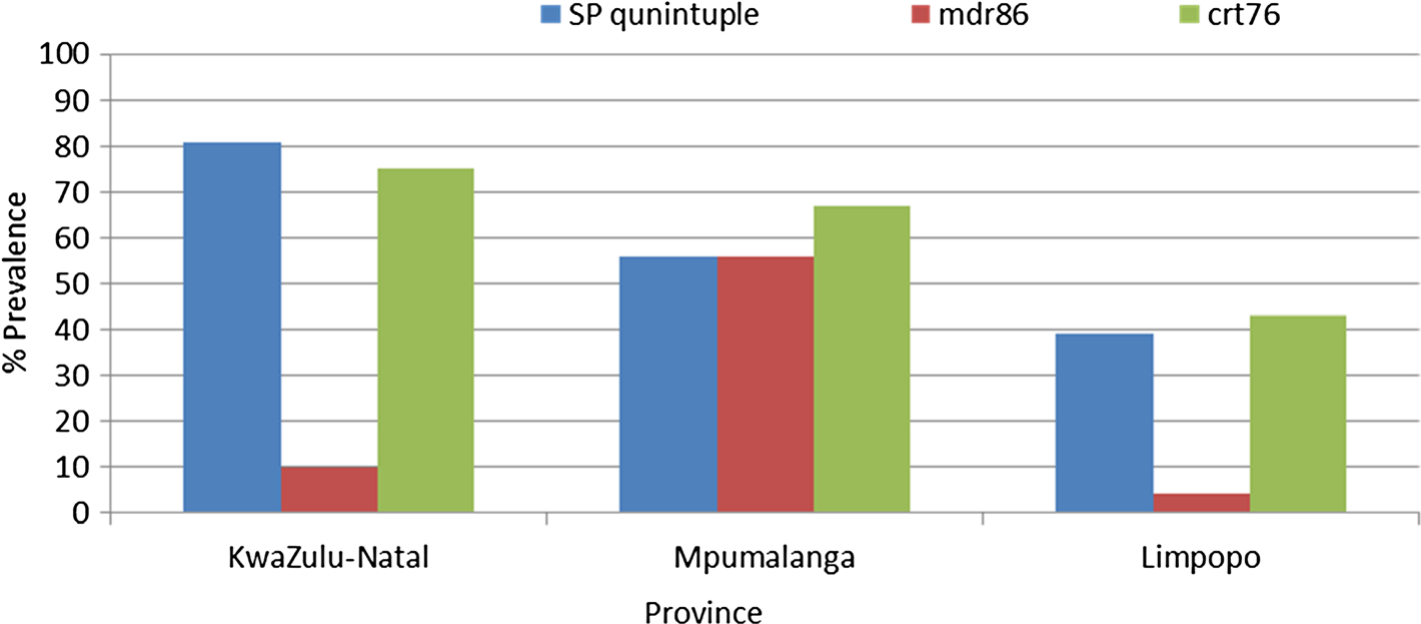
Prevalence of molecular markers associated with chloroquine and sulphadoxine-pyrimethamine resistance in KwaZulu-Natal, Limpopo and Mpumalanga, South Africa in 2010.

By 2010, markers associated with SP and chloroquine resistance were highly prevalent in all three malaria-endemic provinces (Figure 5). Both the SP quintuple and *crt*76 mutations were approaching fixation in KwaZulu-Natal with over 80% of the parasites analysed carrying the quintuple mutation and approximately 73% of the parasite isolates tested mutant at *crt* codon76. Prevalence of the SP and chloroquine resistance markers was the lowest in Limpopo Province, at 38% and 42%, respectively.

While mutations at *mdr1* codon86 were rare in both KwaZulu-Natal (10%) and Limpopo Province (3%), they were rather common in Mpumalanga (Figure 5). More than 50% of the *falciparum* isolates from Mpumalanga carried the mutant *mdr1*86 marker. No variation in *mdr1* copy number was detected in any of the 2010 samples analysed.

## Discussion

Malaria has long been regarded as a major public health burden in South Africa, affecting vast regions of the country. During the late 1920s, malaria epidemics spread as far south as Port St Johns [22], with more than 20,000 deaths reported in a single season [23]. To address the malaria threat, two interventions, residual spraying with insecticides (initially DDT and later pyrethroids) and surveillance were introduced in 1946. These interventions, particularly IRS with DDT resulted in malaria being eliminated from large areas of the country while reducing it to low levels in the three malaria-endemic provinces. Seasonal focal outbreaks associated with favourable climatic conditions and migrant movement, still occurred [24]. The impact of IRS on malaria distribution and cases numbers in South Africa was so dramatic because IRS operations eliminated an extremely efficient malaria vector *An. funestus* and greatly reduced the density and distribution of the main malaria vector *An. arabiensis*. Despite the existence of a very effective, integrated malaria control programme comprising effective vector control and good case management, malaria continues to linger in restricted areas within the malaria-endemic provinces.

Embarking on a malaria elimination agenda is costly, time consuming and extremely labour intensive. It is therefore imperative to ensure the timing of an elimination intervention is appropriate and that all necessary resources are in place as failure would have a deleterious effect on the gains made against malaria transmission. Following 10 years of intensive, integrated, malaria control interventions, malaria incidence in South Africa has declined markedly. The three endemic provinces are at different stages in the malaria elimination continuum, proposed by WHO [6].

Nevertheless, many malaria-endemic districts now meet the incidence limit required to move into the malaria elimination phase. Unfortunately the data from this study show the decrease in malaria incidence has not been uniform across all districts in the malaria-endemic provinces. KwaZulu-Natal is the only province in which all the districts have achieved the minimum elimination incidence level. Therefore only in KwaZulu-Natal can all the efforts be focused solely on malaria elimination. The picture is very different and rather more complex in the remaining two malaria-endemic provinces.

Both Limpopo and Mpumalanga have districts at each of the four categories on the elimination continuum. The border areas of these two provinces are most affected by malaria, with Mozambique largely influencing the malaria incidence in Mpumalanga and the transmission intensity in Zimbabwe impacting on the malaria incidence in Limpopo. Given the wide range of malaria incidence in districts of Limpopo and Mpumalanga, intervention efforts focussing on control and pre-elimination would be most appropriate in these provinces.

In addition to highlighting the complex malaria status pattern, this study found several other areas where information, essential for the formulation of an effective elimination strategy, was lacking. The first area of concern is vector control. Although IRS operations have been rigorously adhered to, the routine assessment of vector distribution and efficacy evaluation of insecticides used for IRS operations have not been conducted in the three malaria-endemic provinces. As no current vector distribution data are available, it is not be possible to meet the elimination requirement of targeted vector control measures.

This issue is further compounded by the lack of current insecticide efficacy data. Data available are over a decade old and more than likely no longer valid given the changes in insecticide pressure over the past 10 years. The emergence of pyrethroid-resistant vectors, a major factor contributing to the 1999/2000 malaria epidemic, led to the re-introduction of DDT-based IRS operations in 2000. This policy change was crucial to controlling the malaria epidemic, particularly in KwaZulu-Natal [25]. While no epidemics have been reported since 2000, DDT resistant *An. arabiensis* populations were found in KwaZulu-Natal in 2003 [18]. Unfortunately no new studies on the emergence and or spread of insecticide resistance have been conducted in South Africa.

In the past five years, all malaria-endemic provinces have reported spray coverage rates greater than 80%, the level of coverage recommended by the WHO to achieve maximum impact. Since the IRS campaign is not achieving the desired outcome (in terms of disease burden reduction) in all provinces, it is possible there are flaws in IRS programme. Possible confounders include the use of incorrect insecticides, poor drug administration, importation of malaria or general programme failures. Further, data for IRS coverage prior to 2005 are not readily available making the assessment of IRS impact prior to 2005 virtually impossible. Although, at present the SIS appears to be functioning effectively, there is a lag period in case reporting which needs to be improved upon in order for case reporting to be made in real-time.

Despite the existence of the MIS in all three malaria-endemic provinces, data collected across all three provinces are not uniform. Even more concerning is the fact that essential data such as quantities of Coartem^®^ dispensed, numbers of RDTs used and numbers of positive smears/RDTs obtained are not being captured by the MIS in any of three provinces. Probable sources of infection were routinely captured by the MIS in Mpumalanga, while it appeared more *ad hoc* in Limpopo and KwaZulu-Natal. Active case detection data were incomplete across all three provinces. These issues need to be addressed urgently as the country has already embarked on an elimination agenda.

Routine anti-malarial resistance marker surveillance across all three malaria-endemic provinces revealed a high prevalence of *P. falciparum* parasites carrying mutant alleles associated with SP and chloroquine resistance. This finding was surprising given the limited current use of both these drugs in South Africa. Chloroquine was replaced by SP as first line treatment in 1991 following reports of growing chloroquine resistance. Sulphadoxine-pyrimethamine remained effective for approximately 10 years before it was phased out of use in KwaZulu-Natal in 2000 and in Mpumalanga and Limpopo in 2003 owing to high levels of SP treatment failures. The resistance data generated by this study supports the South African Department of Health’s decision to replace SP with the ACT, Coartem^®^. On a cautionary note however, given that tolerance to Coartem^®^ has been detected along the Thailand-Myanmar border area [26], it is essential more rigorous sentinel site-based drug resistance surveillance is conducted. Currently, only a small subsets of all positive RDTs are surveyed for the presence of markers associated with lumefantrine resistance. Greater efforts must be made to improve surveillance in this regard.

For the next six years at least (until 2018), it is vital that the South African Government continues to implement its hugely successful malaria control programme while encouraging the creation and roll out of effective control programmes in neighbouring countries, a ground breaking example being the Lubombo Spatial Development Initiative [27]. This regional collaboration between Swaziland, Mozambique and South Africa, aimed at strengthening vector control and case management in these countries, contributed significantly to the substantial reductions in malaria case numbers in southern Mozambique as well as the border areas of KwaZulu-Natal and Mpumalanga. Over the 10 year lifespan of this initiative, malaria prevalence in southern Mozambique declined from over 60% pre-intervention to below 15% by 2011 [28].

Malaria elimination in South Africa is only possible if elimination is also successfully occurring in neighbouring countries, particularly Mozambique and Zimbabwe. The current social, political and economic upheavals in Zimbabwe have contributed to the breakdown of the Zimbabwean malaria control programme. This has resulted in a large number of cases imported from Zimbabwe being reported in Limpopo, preventing the province from attaining a pre-elimination classification.

None the less, South Africa has embarked on programme re-orientation in areas fulfilling the WHO elimination criteria. Vector based interventions, like IRS operations are being scaled down, limited to areas of confirmed local transmission. In malaria endemic areas no longer under control it is hoped that intensive surveillance will elicit rapid outbreak responses in the event malaria cases (local or imported) area detected. The extreme risk associated with re-orientation is severe malaria epidemics in areas were no malaria has been detected for a number of years, particularly if the response alert thresholds are not sensitive enough and/or surveillance not rigorous enough. At present in South Africa there is a great paucity of essential information especially pertaining to the current status of tools being used to implement the elimination agenda [29]. This information is vital for determining the success or failure of the elimination effort and must be collected as soon as possible.

## Conclusions

Data generated by this study suggest that malaria elimination in South Africa is feasible. The IRS programme has successfully decreased malaria transmission, both spatially and temporally. The malaria case fatality rate has been reduced to extremely low levels through the implementation of effective case management protocols. However, there are many gaps in knowledge that must be filled before the country can successfully transition into the WHO defined malaria elimination phase for a sustained period of time. Surveillance of malaria morbidity and mortality, the delivery and use of diagnostic kits and the dispensing and use of ACT needs to be improved. The surveillance for insecticide resistance must become routine across all malaria endemic provinces, while drug resistance surveillance needs to be strengthened in light of decreasing case numbers. The issue of migration and malaria must to be addressed as a matter of priority, particularly as a very large proportion of the malaria cases reported in South Africa are imported from neighbouring countries and the rest of Africa.

It is of paramount importance that financing to achieve and sustain elimination is secured. Underfunding could result in the successful rebounding of malaria as seen in countries like Sri Lanka and India. Crude estimates suggest that the annual costs of achieving elimination in South Africa would be approximately USD6 per person at risk. However, this figure excludes the cost of sustaining elimination which needs to be urgently researched and factored into elimination cost estimates.

These gaps in knowledge and processes are not insurmountable and can be overcome relatively easily with the appropriate research and development of surveillance tools. Malaria elimination in South Africa by 2018 is conceivable provided there is adequate sustainable financing, the appropriate operational research is conducted and there is continued political commitment at all levels, both nationally and internationally.

## Competing interests

The authors declare they have no competing interests.

## Authors’ contributions

RM reviewed the literature, defined the study and drafted the manuscript. JR reviewed the literature, drafted and edited the manuscript and conducted the molecular analyses. RM, JR, IS and NM performed the statistical analysis. NM drafted and edited the manuscript and generated the spatial data and outputs. IS generated the IRS data. PK, DM, AM and ER were involved in data collection and drafting of the manuscript. All authors read and approved the final draft of the manuscript.
